# Silencing Transglutaminase Genes *TGase2* and *TGase3* Has Infection-Dependent Effects on the Heart Rate of the Mosquito *Anopheles gambiae*

**DOI:** 10.3390/insects13070582

**Published:** 2022-06-26

**Authors:** Abinaya Ramakrishnan, Julián F. Hillyer

**Affiliations:** Department of Biological Sciences, Vanderbilt University, Nashville, TN 37235, USA; abinaya.ramakrishnan@vanderbilt.edu

**Keywords:** *Anopheles gambiae*, dorsal vessel, heart, hemocyte, infection, insect, mosquito, ostia, RNAi, transglutaminase

## Abstract

**Simple Summary:**

The immune and circulatory systems of insects are functionally integrated. An infection induces the migration of immune cells called hemocytes to the surface of the heart, where they kill pathogens around valves called ostia. In mosquitoes, a transglutaminase inhibits the infection-induced aggregation of hemocytes on the heart. Here, we studied whether transglutaminases also modify the heart contraction rate. First, we confirmed that an infection decreases the mosquito heart rate. Then, we found that disrupting transglutaminase genes has infection-dependent effects on the heart rate. Silencing *TGase1* does not affect heart physiology. However, silencing *TGase2* eliminates the infection-induced decrease in the heart rate, and silencing *TGase3* decreases the heart rate in uninfected mosquitoes but increases the heart rate in infected mosquitoes. These experiments identify new factors that affect heart physiology in mosquitoes.

**Abstract:**

Transglutaminases are pleiotropic enzymes that in mosquitoes participate in the formation of the mating plug and the wound-induced antimalarial response. Moreover, one transglutaminase, *TGase3*, negatively regulates the infection-induced aggregation of hemocytes on the heart. Given that *TGase3* is an inhibitor of periostial hemocyte aggregation, we used RNAi-based gene silencing followed by intravital video imaging to scrutinize whether any of the three transglutaminases encoded in the genome of the mosquito, *Anopheles gambiae*, play a role in modulating the heart rate of uninfected and infected mosquitoes. Initially, we confirmed that an infection decreases the heart rate. Then, we uncovered that silencing *TGase1* does not impact heart physiology, but silencing *TGase2* results in a constant heart rate regardless of infection status, eliminating the infection-induced decrease in the heart rate. Finally, silencing *TGase3* decreases the heart rate in uninfected mosquitoes but increases the heart rate in infected mosquitoes. We conclude that *TGase2* and *TGase3* modulate heart physiology and demonstrate that factors not classically associated with insect circulatory physiology are involved in the functional integration of the immune and circulatory systems of mosquitoes.

## 1. Introduction

Transglutaminases are calcium-dependent enzymes that crosslink proteins by catalyzing the formation of isopeptide bonds between glutamine and lysine residues [1,2]. In insects, transglutaminases are involved in cuticular morphogenesis, hemolymph coagulation, pathogen entrapment, peritrophic matrix formation, fertility, and immune system modulation [3,4,5,6,7,8]. Additionally, in the fruit fly, *Drosophila melanogaster*, transglutaminase is a negative regulator of the immune deficiency pathway (IMD). Specifically, transglutaminase crosslinks the N-terminal region of the IMD pathway’s transcription factor, Relish, forming a polymer that prevents its translocation into the nucleus, thereby allowing for increased bacterial proliferation following a septic infection [9]. Transglutaminase further inhibits the IMD pathway by incorporating natural polyamines into the DNA binding site of Relish [10]. However, transglutaminase does not always inhibit immunological function. *Drosophila* transglutaminase targets microbial surfaces, and larvae with reduced transglutaminase levels experience decreased survival [11,12]. Moreover, transglutaminase is a positive driver of immune processes in the termite, *Reticulitermes chinensis*, and the silk moth, *Bombyx mori* [13,14].

In mosquitoes, transglutaminases play roles in reproduction and immunity. The genome of the African malaria mosquito, *Anopheles gambiae*, encodes three transglutaminases [15]. The function of *TGase1* is unknown, but *TGase2* positively drives the wound-induced antimalarial response and *TGase3* functions in the formation of the mating plug as well as the infection-induced aggregation of hemocytes on the heart [15,16,17,18,19]. Therefore, from an immunological perspective, *TGase3* interfaces with the circulatory system.

The immune and circulatory system of mosquitoes—and insects in general—are functionally integrated [20,21,22]. An infection induces the migration of hemocytes to the surface of the heart, where they become sessile and phagocytose pathogens around heart valves called ostia. These heart-associated hemocytes, called periostial hemocytes, are advantageous because they attack circulating pathogens in locations of high hemolymph flow [23]. Although the structural mechanics of periostial hemocyte aggregation have been defined, the molecular drivers of this process are less understood. Members of the thioester containing protein family (TEP) and nimrod protein family (NIM) influence periostial hemocyte aggregation [24,25]. Moreover, the IMD pathway and the interlinked JNK pathway—as tested via the RNAi-based silencing of the positive regulators *rel2*, *JNK1* and *JNK3*, and the negative regulators *caspar* and *puckered*—drive periostial hemocyte aggregation [26]. An RNAseq analysis in that same study identified transglutaminases as being more highly expressed in the heart containing periostial hemocytes than in other tissues [26], and follow-up experiments confirmed that *TGase3*—but not *TGase1* or *TGase2*—is a negative regulator of periostial hemocyte aggregation during the early stages of infection, presumably by inhibiting the IMD pathway [19]. However, whether transglutaminases affect how the heart contracts remained unknown. This question is of particular interest because a systemic infection reduces the mosquito heart rate, and this is modulated by nitric oxide produced by periostial hemocytes [27].

The main circulatory organ in mosquitoes is the dorsal vessel, which is a muscular tube that traverses the length of the body and is subdivided into an aorta in the thorax and a heart in the abdomen [28,29,30,31]. Hemolymph enters the dorsal vessel through ostia that are present in every abdominal segment and is then propelled by wave-like contractions of the heart muscle [28,29]. The mosquito heart is myogenic, but its rhythmicity can be altered by both endogenous and exogenous factors [27,32,33,34,35,36]. In this study, we scrutinized whether RNAi-based silencing of transglutaminases affects the mosquito heart rate in both uninfected and infected mosquitoes. We uncovered that *TGase3* silencing and, to a lesser extent *TGase2* silencing, has infection-dependent effects on the mosquito heart rate.

## 2. Materials and Methods

### 2.1. Mosquito Rearing, Maintenance and Manipulation

Experiments were conducted on female *Anopheles gambiae*, Giles sensu stricto (G3 strain; Diptera: Culicidae). Mosquitoes were reared and maintained in an environmental chamber at 27 °C and 75% relative humidity, with a 12 h:12 h light–dark photoperiod [36]. Upon emergence, adults were fed ad libitum on cotton pads saturated with 10% sucrose. Prior to a manipulation, mosquitoes were anesthetized by placing them in a Petri dish resting over ice, and injections into the hemocoel were administered at the thoracic anepisternal cleft using a Nanoject III Programmable Nanoliter Injector (Drummond Scientific Company, Broomall, PA, USA).

### 2.2. Synthesis of Double-Stranded RNA (dsRNA) and RNA Interference (RNAi)

Double-stranded RNA was synthesized for three mosquito genes—*TGase1* (AGAP009100), *TGase2* (AGAP009098) and *TGase3* (AGAP009099)—and one control bacterial gene—*bla(Ap^R^)*—using methods previously described [19]. Specifically, gene-specific primers with T7 promoter tags (Table 1) were used to amplify a fragment of each gene using as a template cDNA from *A. gambiae* for transglutaminase genes or DNA from *E. coli* BL21(DE3) containing the pET-46 plasmid (EMD Chemicals, Gibbstown, NJ, USA) for the bacterial gene. The amplicons were subjected to agarose gel electrophoresis, and the bands of interest were excised and purified using Qiagen’s QIAquick Gel Extraction kit. The purified amplicons were then used as the template in a second PCR reaction with the same primers. The product of this reaction was purified using the QIAquick PCR Purification Kit (Qiagen, Valencia, CA, USA), and up to 1 μg of the purified product was used as the template for in vitro dsRNA synthesis using the MEGAscript T7 Kit (Applied Biosystems, Foster City, CA, USA). The dsRNA was precipitated using ethanol and re-suspended in phosphate-buffered saline (PBS). The dsRNA concentration was quantified spectrophotometrically using a BioPhotometer Plus spectrophotometer (Eppendorf AG, Hamburg, Germany), and the integrity of the dsRNA was verified by gel electrophoresis. To initiate RNAi, two-day-old female mosquitoes were injected with 300 ng of a dsRNA.

### 2.3. Mosquito Treatments: Uninfected and Infected with E. coli

Four days after dsRNA injection (i.e., at 6 days of adulthood), mosquitoes were subjected to one of three treatments: (i) unmanipulated (called the uninfected condition), (ii) infected with *E. coli* for 4 h, or (iii) infected with *E. coli* for 24 h. Mosquito infections were conducted using tetracycline resistant GFP-expressing *E. coli* (modified DH5α). These bacteria were grown overnight in Luria–Bertani’s (LB) rich nutrient medium in a 37 °C shaking incubator (New Brunswick Scientific, Edison, NJ, USA). Infection was initiated by injecting 69 nL of a dilution of the bacterial culture into the hemocoel, and the infection dose was determined by plating dilutions of the inoculum on LB agar plates and counting the colony-forming units that grew on the plates. On average, mosquitoes were infected with 15,374 GFP-*E. coli*. An injury group, such as a mock injection, was omitted because injury does not induce periostial hemocyte aggregation [20,22,23].

### 2.4. cDNA Synthesis and Quantitative RT-PCR (qPCR) for RNAi Efficiency Determination

RNA was extracted and isolated from a pool of 10 whole-body mosquitoes for each dsRNA-treatment combination. RNA was initially purified using TRIzol Reagent (Applied Biosystems, Waltham, MA, USA) and then re-purified using the RNeasy Mini Kit (Qiagen, Valencia, CA, USA). The RNA concentration was measured spectrophotometrically, and up to 5 μg of RNA was treated with RQ1 RNase-free DNase (Promega, Madison, WI, USA) and subsequently used for cDNA synthesis using Oligo(dT)_20_ primers and the SuperScript III First-Strand Synthesis System for RT-PCR (Applied Biosystems).

qPCR was conducted using cDNA as a template, gene-specific primers (Table 1), and Power SYBR Green PCR Master Mix (Applied Biosystems) on a CFX Connect Real-Time Detection System (Bio-Rad Laboratories, Hercules, CA, USA). The 2^−ΔΔC^_T_ method was used for relative quantification, using the housekeeping gene *RpS7* as the reference [36,37,38]. The mRNA fold change for each transglutaminase gene (indicative of silencing efficiency) was calculated relative to ds*bla(Ap^R^)* mosquitoes of the same treatment. For each dsRNA-treatment combination, 2–3 independent trials were conducted, each with two technical replicates. These trials were conducted on some of the same mosquitoes for which heart physiology measurements were made. That is, mosquitoes were placed in TRIzol immediately after the acquisition of a video.

### 2.5. Measurement of Heart Contractions

Mosquitoes were placed on Sylgard 184 silicone elastomer plates (Dow Corning, Midland, MI, USA) with wings spread and ventral side down using a noninvasive procedure we have described and pictured [28,39]. After mosquitoes acclimated to room temperature, their dorsal abdomens—and the underlying heart—were viewed under bright-field trans-illumination using a Nikon SMZ1500 stereo microscope (Nikon Corporation, Tokyo, Japan) connected to a Hamamatsu ORCA-Flash 2.8 digital CMOS camera (Hamamatsu Photonics, Hamamatsu, Japan) and Nikon Advanced Research NIS-Elements software. Videos of 65 s duration were recorded, and the middle 60 s of each video were manually analyzed for heart contractions, leaving buffers of 2.5 s at the beginning and end of each video. The heart contraction rate in hertz (Hz, or contractions per second) was determined by counting the frequency of wave-like contractions of cardiac muscle, as seen through the semi-translucent abdomen [28,40]. For both the uninfected and the 24 h infection conditions, 30–40 mosquitoes were assayed per dsRNA treatment across 4 independent biological trials. For the 4 h infection condition, 15–20 mosquitoes were assayed per treatment across 2 biological trials. Some of the 24 h trials were conducted on the same mosquito batches as the 4 h trials, so some of the uninfected mosquito readings are used when reporting both the 24 h and 4 h data. Heart contraction data were analyzed by two-way ANOVA followed by Sidak’s multiple comparisons tests, using GraphPad Prism version 9.3.1. The data collected for this manuscript is contained in the Supplementary Materials (Data File S1).

## 3. Results

### 3.1. General Experimental Design and the Efficiency of RNAi-Based Silencing of Transglutaminases

Two-day-old female mosquitoes were divided into four RNAi groups: (i) ds*bla(Ap^R^)*, (ii) ds*TGase1*, (iii) ds*TGase2*, and (iv) ds*TGase3*. Four days later (at 6 days of adulthood), each dsRNA group was divided into two treatment conditions: (i) uninfected and (ii) infected with *E. coli*. Each infected condition was assayed at 24 h and 4 h after treatment.

**Figure 1:**
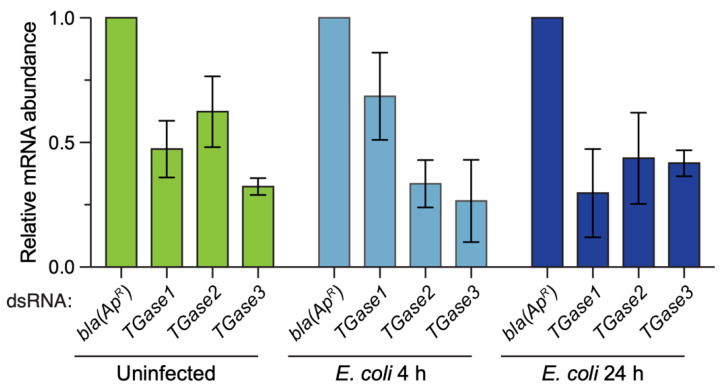
RNAi-based knockdown efficiency of *TGase1*, *TGase2* and *TGase3* in uninfected and infected mosquitoes. Graphs display the relative mRNA abundance in mosquitoes treated with *bla(Ap^R^)*, *TGase1*, *TGase2* or *TGase3* dsRNA. Mosquitoes were uninfected or had been infected with *E. coli* for 4 or 24 h, and mRNA abundance was calculated relative to the *bla(Ap^R^)* for each treatment. Column heights mark the mean, and whiskers indicate the standard error of the mean (S.E.M.).

To ensure that the dsRNA injections were impacting the mRNA abundance of the target genes, we determined the efficiency of RNAi by qPCR (Figure 1). RNAi efficiency varied depending on the treatment–time combination, but on average, silencing of *TGase1* was 52%, *TGase2* was 54% and *TGase3* was 67%. This RNAi efficiency is consistent with our earlier work silencing transglutaminases [19]. 

### 3.2. RNAi-Based Silencing of Transglutaminases Has Infection-Dependent Effects on the Heart Rate at 24 h after Treatment

**Figure 2:**
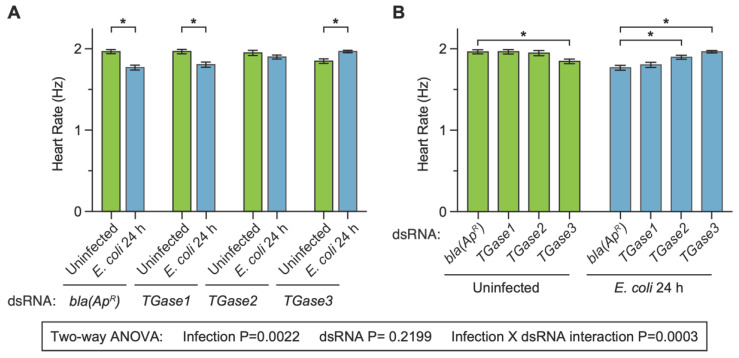
Effect of RNAi-based silencing of *TGase1*, *TGase2* or *TGase3* on the heart rate at 24 h after treatment. Graphs show the average heart contraction rates in mosquitoes treated with *bla(Ap^R^)*, *TGase1*, *TGase2* or *TGase3* dsRNA, and were either uninfected or infected with *E. coli* for 24 h. The same data are presented in two configurations: panel (**A**) groups the data by dsRNA treatment whereas panel (**B**) groups the data by infection status. Data were analyzed by two-way ANOVA followed by Sidak’s post hoc test. Column heights mark the means, and whiskers show the S.E.M. Asterisks (*) indicate post hoc *p* < 0.05.

We began assessing whether transglutaminase silencing affects the heart rate in uninfected and infected mosquitoes at 24 h post-treatment because we have previously observed that an infection decreases the heart rate at 1-, 3- and 5-days post-infection [27]. The heart of ds*bla(Ap^R^)* uninfected mosquitoes contracted at 1.96 Hz, and infection for 24 h decreased the heart contraction rate by 9.9% (Figure 2A). This infection-induced decrease is similar to what we have previously observed following infection with bacteria [27]. Similar to ds*bla(Ap^R^)* mosquitoes, infection reduced the heart contraction rate of ds*TGase1* mosquitoes by 8.2%. However, the infection-induced decrease in the heart rate disappeared in ds*TGase2* mosquitoes (−0.8% difference) and was reversed to an infection-induced increase of 6.4% in ds*TGase3* mosquitoes.

When we instead compared the effect of injecting different types of dsRNAs within a single treatment (comparing the effect of dsRNA within the uninfected, or within 24 h after infection), additional patterns emerged (Figure 2B). The heart rate of uninfected mosquitoes that had been treated with ds*bla(Ap^R^)*, ds*TGase1* and ds*TGase2* were all similar (<0.8% difference from control), but the heart rate of uninfected ds*TGase3* mosquitoes was 6% lower than that of ds*bla(Ap^R^)* mosquitoes. At 24 h after infection, the heart rate of ds*bla(Ap^R^)* and ds*TGase1* mosquitoes was similar (2% difference), but relative to infected ds*bla(Ap^R^)* mosquitoes, the heart rate of infected ds*TGase2* and ds*TGase3* mosquitoes was elevated by 7.3% and 11.1%, respectively.

Statistical analysis of the entire 24 h dataset revealed that infection status irrespective of the type of dsRNA affects the heart rate (*p* = 0.0022) but that the type of dsRNA irrespective of the infection status does not (*p* = 0.2199). Moreover, there was a significant interaction between infection status and dsRNA treatment (*p* = 0.0003), indicating that the effect of infection changes when different transglutaminases are silenced by RNAi (and vice versa). Sidak’s multiple comparisons tests then revealed that ds*bla(Ap^R^)* and ds*TGase1* mosquitoes display the expected infection-induced reduction in the heart rate, but that this effect disappears in ds*TGase2* mosquitoes and is reversed in ds*TGase3* mosquitoes. In other words, there is a dichotomy in how silencing different transglutaminases affects the heart rate in uninfected and infected mosquitoes.

### 3.3. The Infection-Dependent Cardiac Effect of Silencing Transglutaminases Is Recapitulated at 4 h after Treatment

**Figure 3:**
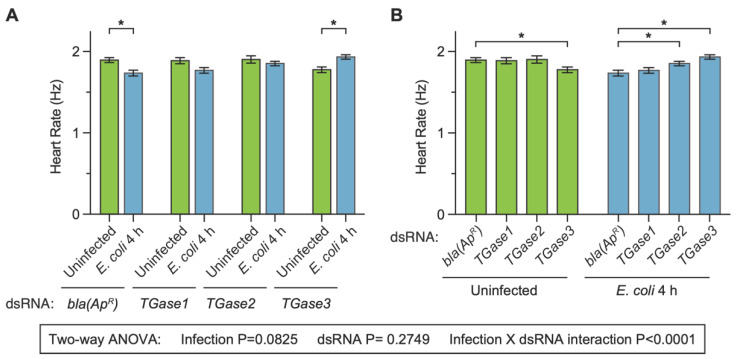
Effect of RNAi-based silencing of *TGase1*, *TGase2* or *TGase3* on the heart rate at 4 h after treatment. Graphs show the average heart contraction rates in mosquitoes treated with *bla(Ap^R^)*, *TGase1*, *TGase2* or *TGase3* dsRNA, and were either uninfected or infected with *E. coli* for 4 h. The same data are presented in two configurations: panel (**A**) groups the data by dsRNA treatment whereas panel (**B**) groups the data by infection status. Data were analyzed by two-way ANOVA followed by Sidak’s post hoc test. Column heights mark the means, and whiskers show the S.E.M. Asterisks (*) indicate post hoc *p* < 0.05.

After determining that transglutaminase silencing has infection-dependent effects on the mosquito heart rate at 24 h following treatment, we proceeded to test whether this effect was recapitulated at 4 h following treatment. We did so because we recently showed that silencing *TGase3*—but not *TGase1* or *TGase2*—affects the infection-induced aggregation of hemocytes on the surface of the heart at 4 h after treatment [19]. The heart of ds*bla(Ap^R^)* uninfected mosquitoes contracted at 1.89 Hz, and infection for 4 h decreased the heart contraction rate by 8.4% (Figure 3A). Again, this infection-induced decrease in the heart rate is similar to what we have previously observed following infection with bacteria [27], and it is also similar to what we observed here at 24 h following treatment (Figure 2). Similar to ds*bla(Ap^R^)* mosquitoes, infection decreased the heart rate of ds*TGase1* mosquitoes by 6.3%. However, the infection-induced decrease in the heart rate of ds*TGase2* mosquitoes was attenuated to 2.6% and reversed in ds*TGase3* mosquitoes; the heart rate of ds*TGase3* mosquitoes increased by 8.9% at 4 h after infection relative to uninfected ds*TGase3* mosquitoes.

When we compared the effect of injecting different types of dsRNAs within a single immune treatment, we found that the heart rate of ds*bla(Ap^R^)* and ds*TGase1* mosquitoes that were uninfected was nearly identical (−0.4% difference), as was their heart rate at 4 h after infection (1.9% difference) (Figure 3B). The heart rate of ds*bla(Ap^R^)* and ds*TGase2* mosquitoes that were uninfected was also nearly identical (0.4% difference), but after 4 h of infection, the heart rate of ds*TGase2* mosquitoes was 6.8% higher than that of infected ds*bla(Ap^R^)* mosquitoes. Finally, the heart rate of ds*TGase3* uninfected mosquitoes was 6.3% lower than that of ds*bla(Ap^R^)* uninfected mosquitoes, yet this phenotype reversed after infection; the heart rate at 4 h post-infection was 11.4% higher in ds*TGase3* mosquitoes than in ds*bla(Ap^R^)* mosquitoes.

Two-way ANOVA revealed that, across the entire 4 h dataset, infection status irrespective of the type of dsRNA does not affect the heart rate (*p* = 0.0825), and neither does the type of dsRNA irrespective of infection status (*p* = 0.2749). However, there was a significant interaction between infection status and the type of dsRNA (*p* < 0.0001), once again indicating that the effect of infection changes when different transglutaminases are silenced by RNAi (and vice versa). The post hoc tests revealed that ds*bla(Ap^R^)* mosquitoes—and perhaps ds*TGase1* mosquitoes—display the expected infection-induced reduction in the heart rate, but that this effect is lost in ds*TGase2* mosquitoes and reversed in ds*TGase3* mosquitoes. Overall, the data collected at 4 h and 24 h are in agreement with one another. Moreover, the expected infection-induced decrease in the heart rate is evident in control mosquitoes and mosquitoes where *TGase1* was disrupted but not in mosquitoes where either *TGase2* or *TGase3* was disrupted.

## 4. Discussion

**Figure 4:**
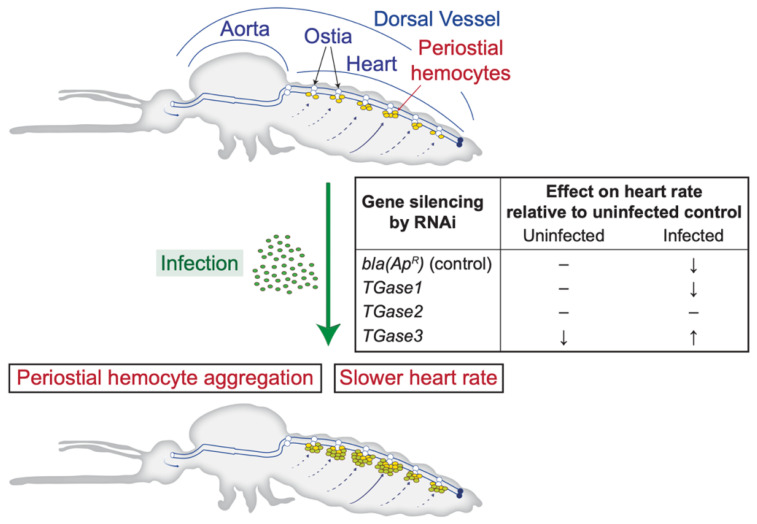
Diagrammatic representation of the mosquito circulatory system and the process of periostial hemocyte aggregation, together with the effect of silencing transglutaminase genes on the heart contraction rate of uninfected and infected mosquitoes.

Infection decreases the heart rate, and this is modulated by nitric oxide produced by heart-associated hemocytes [27,41,42]. Although multiple factors affect heart contraction dynamics [43,44,45], whether regulators of periostial hemocyte aggregation affect the heart rate remained unknown. In the present study, we confirmed that an infection decreases the heart rate and uncovered that *TGase3*—and to a lesser extent *TGase2*—affect the heart rate in an infection-dependent manner. Specifically, silencing *TGase1* has no impact on heart physiology and recapitulates that an infection decreases the heart rate. Silencing *TGase2* leads to a constant heart rate regardless of whether a mosquito is infected, with the heart rate always being similar to that of an uninfected mosquito; in other words, the infection-induced decrease in the heart rate does not occur. Finally, silencing *TGase3* decreases the heart rate in uninfected mosquitoes but increases the heart rate in infected mosquitoes (Figure 4).

Prior to the initiation of this study, one of our working hypotheses was that neither *TGase1* nor *TGase2* plays a role in heart physiology, and we based this hypothesis on our earlier finding that these genes do not affect periostial hemocyte aggregation [19]. Our other working hypothesis was that *TGase3* does not play a role in heart physiology in uninfected mosquitoes but dampens the infection-induced reduction in the mosquito heart rate; we based this hypothesis on our earlier finding that *TGase3* does not affect periostial hemocyte aggregation in uninfected mosquitoes but negatively affects this process following infection [19]. The hypothesis on *TGase1* is supported by the data but the hypotheses on *TGase2* and *TGase3* are not. The phenotypes observed are difficult to explain, but below, we present several plausible explanations, which are not mutually exclusive.

A possible explanation as to why the heart rate of *TGase3*- or *TGase2*-silenced mosquitoes increases following infection (relative to infected mosquitoes injected with control dsRNA) pertains to the infection-induced deposition of melanin on the heart. Melanization is a multifunctional biochemical cascade driven by phenoloxidase and other enzymes that is essential in the immune response against bacteria and parasites [46,47]. Transglutaminase and prophenoloxidase are both produced by hemocytes, and hemolymph clot formation involves both transglutaminase and phenoloxidase [7,48,49]. Moreover, activation of the melanization cascade in mosquitoes leads to the accumulation of melanin in the periostial regions of the heart [20,23,25,50,51]. We previously observed that silencing *TGase3*, and to a lesser extent *TGase2*, leads to a large increase in melanin deposition within the periostial regions of infected mosquitoes [19]. This melanin could partially block hemolymph entry into the heart via the ostia, and to compensate for the decrease in stroke volume, the heart of these mosquitoes may contract faster. A problem with this explanation is that it does not explain the heart rate phenotype for *TGase3*-silenced uninfected mosquitoes, or *TGase3*- or *TGase2*-silenced mosquitoes that had been infected for 4 h; there is negligible melanin on the heart of those mosquitoes. However, the observation of a more tepid phenotype when silencing *TGase2* when compared to *TGase3* could be due to *TGase3* being more highly expressed in the heart with periostial hemocytes than *TGase2*, as uncovered in our RNAseq screen.

Another potential explanation pertains to downstream effects of the IMD pathway. The genome of *D. melanogaster* encodes one transglutaminase, and it is a negative regulator of the IMD pathway [9,10]. In mosquitoes, *TGase3* is a negative regulator of periostial hemocyte aggregation [19], and because the IMD pathway is a positive regulator of periostial hemocyte aggregation [26], we hypothesized that *TGase3* impacts periostial hemocyte aggregation by inhibiting the IMD pathway. The IMD pathway produces an array of antimicrobial peptides [52,53,54], and therefore, removing an inhibitor of the IMD pathway may increase antimicrobial peptide production. More antimicrobial peptides may quell an infection more quickly, which would decrease the need for nitric oxide. Nitric oxide is an antimicrobial produced by many tissues—including periostial hemocytes—and also reduces the heart rate [27,55,56,57,58], and so, a reduction in nitric oxide may eliminate or dampen the infection-induced decrease in the heart rate. Again, this explanation would be consistent with our data from infected mosquitoes, but it would not explain the phenotype observed in uninfected mosquitoes with silenced *TGase3*.

Other possibilities rely on comparisons with vertebrate animals. For example, transglutaminase causes age-associated vascular stiffening and dysfunction in mice, resulting in a loss of vascular compliance as this mammal ages [59]. It is possible that silencing mosquito *TGase3* reduces heart stiffening, thereby increasing cardiac efficiency and reducing the heart rate in uninfected mosquitoes. Although this does not explain the phenotype observed in *TGase3*-silenced mosquitoes that were infected, it is in agreement with the progressive heart rate increase that takes place as a mosquito ages [32].

## 5. Conclusions

Here, we analyzed the entire transglutaminase gene family of *A. gambiae* in the context of the circulatory system. We conclude that *TGase2* and *TGase3* modulate heart physiology in ways that depend on the infection status of the mosquito and demonstrate that factors not classically associated with circulatory physiology are involved in the functional integration of the immune and circulatory systems of mosquitoes.

## Figures and Tables

**Table 1:** Gene names, gene IDs, and primers used in this study.

Gene	VectorBase ID ^a^	Application	Sequence (Forward and Reverse) ^b^	Amplicon (bp) ^c^	
				Transcript	Genomic
*RpS7*	AGAP010592	qPCR	GACGGATCCCAGCTGATAAA	132	281
			GTTCTCTGGGAATTCGAACG		
*TGase1*	AGAP009100	qPCR	CTGCACAAGGGACTGTTCCA	191	259
			AACGCCAAAAAGCCATCCAC		
*TGase2*	AGAP009098	qPCR	CGGTGGACGCTGACTATCAA	225	297
			GTACTGGCCGAGCTTCCATT		
*TGase3*	AGAP009099	qPCR	TACAGCAGCCAGCGGTTTAG	236	236
			ATATCGCGCCCAGTGTAGTC		
*bla(Ap^R^)*	(Bacterial gene)	RNAi	TAATACGACTCACTATAGGGCCGAGCGCAGAAGTGGT	214	214
			TAATACGACTCACTATAGGGAACCGGAGCTGAATGAA		
*TGase1*	AGAP009100	RNAi	TAATACGACTCACTATAGGGCATTCCGGTTAATCAGT	361	433
			TAATACGACTCACTATAGGGCGTAGTCGATTGTAAGA		
*TGase2*	AGAP009098	RNAi	TAATACGACTCACTATAGGGTCAGAGCTGTCTAACAAA	490	490
			TAATACGACTCACTATAGGCGTACCGCTCAACTCC		
*TGase3*	AGAP009099	RNAi	TAATACGACTCACTATAGGGAAAACCTTCCACACGTC	501	501
			TAATACGACTCACTATAGGGTTGAACAGCACAAACAA		

^a^ Vectorbase IDs were obtained from the AgamP4 assembly in www.vectorbase.org (exception: *bla(Ap^R^)*) (website last accessed 1 June 2022). ^b^ Underlined sequences are specific to the T7 RNA polymerase promoter sites needed for dsRNA synthesis. ^c^ Amplicon sizes are based on the sequences in Vectorbase. For dsRNA primers, amplicon sizes include the T7 promoter sequence tags.

## Data Availability

Data are contained within the article and the Supplementary Material.

## Supplementary Materials

The following supporting information can be downloaded at https://www.mdpi.com/article/10.3390/insects13070582/s1, Data File S1.
